# Selective Silencing of TDP-43 P. G376D Mutation Reverses Key Amyotrophic Lateral Sclerosis-Related Cellular Deficits

**DOI:** 10.3390/biom16030393

**Published:** 2026-03-05

**Authors:** Roberta Romano, Giorgia Ruotolo, Francesco Perrone, Silvia Tomaselli, Martina Mazzoni, Rossella Spataro, Francesca Luisa Conforti, Jessica Rosati, Cecilia Bucci

**Affiliations:** 1 Department of Experimental Medicine, University of Salento, 73100 Lecce, Italy; roberta.romano@unisalento.it; 2 Cell Reprogramming Unit, Fondazione IRCCS Casa Sollievo della Sofferenza, 71013 San Giovanni Rotondo, Italy; g.ruotolo@operapadrepio.it (G.R.); s.tomaselli@operapadrepio.it (S.T.); m.mazzoni@operapadrepio.it (M.M.); j.rosati@operapadrepio.it (J.R.); 3 Department of Biotechnology and Biosciences, University of Milano-Bicocca, 20126 Milan, Italy; 4 Department of Pharmacy, Health and Nutritional Sciences, University of Calabria, 87036 Rende, Italy; francesco.perrone@unical.it (F.P.); francescaluisa.conforti@unical.it (F.L.C.); 5 Intensive Neurorehabilitation Unit, Villa delle Ginestre Hospital, 90135 Palermo, Italy; rossellaspataro@libero.it; 6 Departmental Faculty of Medicine, UniCamillus-Saint Camillus International University of Health Sciences, 00131 Rome, Italy

**Keywords:** RNA interference, ALS, TARDBP, motor neuron

## Abstract

Amyotrophic lateral sclerosis (ALS) is a neurodegenerative disease for which there is currently no cure. Dominant mutations in the *TARDBP* gene are causative of ALS. In particular, the p. G376D substitution in TDP-43 causes familial ALS and it is associated with TDP-43 mislocalization in the cytosol, increased presence of cytoplasmic aggregates, and lysosomal and mitochondrial dysfunction. We previously designed a small interfering RNA (siRNA) that specifically targets and silences the mutant allele and we demonstrated that, in patient-derived fibroblasts, it can reduce TDP-43 aggregation, decrease oxidative stress, and improve cell viability. Here, we investigated the ability of this siRNA to revert some ALS-associated pathological phenotypes in motor neurons derived from induced pluripotent stem cells (iPSCs), as motor neurons are the primary cells affected in ALS. siRNA treatment reduced TDP-43 mislocalization, enhanced lysosomal function and cell viability, and decreased oxidative stress. These findings indicate that this allele-specific siRNA effectively reverses key ALS-related cellular deficits in motor neurons, representing a promising candidate for targeted therapy in patients carrying the TDP-43 G376D mutation.

## 1. Introduction

Amyotrophic lateral sclerosis (ALS) is a fatal neurodegenerative disease affecting upper and lower motor neurons in the motor cortex, brainstem, and spinal cord [1]. The main symptoms of this neurodegenerative disorder are muscle weakness, dysphagia, dysarthria, spasticity, and paralysis [2]. Unfortunately, patients have an average life expectancy between 2 and 5 years after the onset of symptoms, but half of them die within 3 years. This bad prognosis is due to the rapid degeneration of motor neurons, which causes paralysis and death, which often occurs due to respiratory failure [3,4]. The clinical picture is made more heterogeneous by other symptoms, such as cognitive and behavioral changes that may occur [5].

The majority of ALS cases are sporadic (about 90%). In comparison, about 10% of cases are familial, with a genetic cause, usually inherited as an autosomal dominant trait [6,7]. More than 50 genes have been associated with ALS. The most common genes mutated in familial ALS cases are *SOD1* (superoxide dismutase 1), *C9orf72* (chromosome 9 open reading frame 72), *TARDBP* (TAR DNA-binding protein), and *FUS* (fused in sarcoma) [8,9].

3–5% of cases of familial ALS and about 1% of idiopathic ALS are caused by mutations in *TARDBP* [10,11]. This gene encodes TDP-43, a protein that regulates various aspects of RNA metabolism, including transcription, splicing, and transport. In ALS, mutations in TDP-43 are responsible for its mislocalization. Indeed, TDP-43 is predominantly a nuclear protein that can shuttle between the nucleus and the cytoplasm, thanks to the presence of a nuclear localization signal and a nuclear export signal [12,13]. When TDP-43 is mutated, its cytoplasmic presence increases, leading to the formation of insoluble, ubiquitinated aggregates, a hallmark of ALS. This altered localization affects cellular homeostasis, leading to neuronal death [4,14,15]. Indeed, autophagy is altered, stress granule formation is increased, and mitochondria are dysfunctional following TDP-43 aggregate accumulation [16,17,18].

TDP-43 inclusions characterize approximately 97% of patients with familial or sporadic ALS, not just those affected by the disease due to the mutation in the *TARDBP* gene [19]. This makes TDP-43 a major protein signature for ALS [6].

Over 80 ALS-associated mutations have been identified in the *TARDBP* gene. It is worth noting that several of these variants affect the C-terminal region of the TDP-43 protein, an intrinsically disordered domain containing multiple aggregation-prone motifs [10,20]. Mutated TDP-43 is hyperphosphorylated, ubiquitinated, and proteolytically cleaved into C-terminal fragments (CTFs) of ∼25 kDa that form insoluble and toxic aggregates [21,22].

One of these mutations has been identified in an Italian family with several members affected by ALS. It is the heterozygous missense mutation c.1127G>A in Exon 6 of the *TARDBP* gene, responsible for the amino acid substitution glycine-aspartic acid in the 376 position of the protein sequence [23,24]. The presence of this variant is associated with TDP-43 mislocalization leading to the formation of cytoplasmic inclusions [25]. This leads to cellular stress, with decreased cellular viability and dysfunction of several organelles, such as lysosomes and mitochondria. Indeed, ALS cells carrying the TDP-43 G376D mutation are characterized by reduced lysosomal acidification and degradative activity [26]. Moreover, ALS cells exhibit fragmented mitochondria, characterized by reduced mitochondrial membrane potential and alterations in the mitochondrial respiratory chain. Additionally, TDP-43 G376D is associated with reduced abundance of antioxidant enzymes, leading to increased oxidative stress [27]. The treatment of the disease is challenging due to the wide variety of genes contributing to the development of ALS. Riluzole (6-(trifluoromethoxy)-2-aminobenzothiazole) has been the only treatment available for ALS patients for a long time, and its mechanism of action is based on the reduction in glutamate excitotoxicity [28,29]. More recently, Edavarone has been approved and acts as an antioxidant and free-radical scavenger [30,31]. More recently, two drugs have been shown to be helpful in reducing endoplasmic reticulum and mitochondrial dysfunction, diminishing neuronal apoptosis and oxidative stress: Sodium Phenylbutyrate and Taurursodiol [32,33]. However, all these medications only marginally slow the progression of the disease.

Considering the lack of effective treatments, new therapeutic approaches are necessary. The focus is increasingly shifting towards gene therapy. An antisense oligonucleotide, Tofersen, has been approved for the treatment of SOD1-related ALS cases. It has demonstrated therapeutic effectiveness in reducing SOD1 levels, resulting in reduced neuronal damage and slowed disease progression [34,35].

The scenario is more complex in the case of TDP-43, considering that the levels of this protein have to be tightly regulated. Indeed, neuronal cells suffer whether there is an increase or a decrease in TDP-43 expression [36]. A strategy could involve silencing the mutant allele by leveraging TDP-43’s ability to regulate its own levels [37]. Recently, we identified a siRNA (called m10) complementary to the region comprising the point mutation c.1127G>A and that targets explicitly the TDP-43 mutant allele. Indeed, transfected cells expressing the TDP-43^G376D^ mutant protein or skin-derived fibroblasts from ALS patients carrying the TDP-43^G376D^ mutation exhibited reduced TDP-43 mislocalization, lower cytoplasmic accumulation of TDP-43, reduced oxidative stress, and increased cell viability following treatment with m10. Therefore, m10 has proven to be effective in reversing some pathological phenotypes in these cellular models [25]. However, motor neurons are the primary cells affected in ALS. Therefore, this study aims to evaluate the effectiveness of m10 in correcting the disease-associated phenotype in induced pluripotent stem cell (iPSC)-derived motor neurons carrying the G376D mutation in TDP-43.

## 2. Materials and Methods

### 2.1. Induced Pluripotent Stem Cells Generation and Motor Neurons Differentiation

We differentiated three iPSC lines in motor neurons. iPSCs were obtained by reprogramming skin biopsy-derived fibroblasts from a healthy control and an ALS patient carrying the p.G376D TDP-43 mutation. This patient underwent a skin biopsy at the time of the ALS diagnosis (ALS1O) and four years later (ALS1A). These three stable iPSC lines have already been fully characterized [38]. They were nucleofected with epB-Bsd-TT-NIL and the piggyBac transposase plasmid following the protocol described by Garone et al. [39]. Colonies with integrated piggybacs (NIL-iPSCs) were selected with blasticidin and amplified in Nutristem supplemented with 0.5% of Penicillin-Streptomicin. NIL-iPSCs were plated on Matrigel in a 6-well plate at a density of 80,000 cells/cm^2^ for a week. Then they were enzymatically dissociated with Accutase, and plated on cultrex-coated glass coverslips at a density of 70,000 cells/coverslip. Motor neuron differentiation was maintained up to day 18.

### 2.2. Transfection

At day 12 of differentiation, motor neurons were treated with control RNA or m10 siRNA using Neuromag (OZ Bioscience, San Diego, CA, USA), following the manufacturer’s instructions. m10 siRNA is complementary to the region of the human TDP-43 mRNA comprising the point mutation c.1127G>A responsible for the amino acidic substitution G376D in the protein sequence. The siRNAs were purchased from Eurofins Genomics (Ebersberg, Germany), and their sequences are the following:

Control RNA (as negative control):
sense: 5′-ACUUCGAGCGUGCAUGGCUTT-3′
antisense: 5′-AGCCAUGCACGCUCGAAGUTT-3′
TDPi (a siRNA targeting the 5′ region of TDP-43 mRNA):
sense: 5′-AAGCAAAGCCAAGAUGAGCCUTT-3′
antisense: 5′-UUCGUUUCGGUUCUACUCGGATT-3′
m10:
sense: 5′-CUUAUAGUGACUCUAAUUCTT-3′;
antisense: 5′-GAAUUAGAGUCACUAUAAGTT-3′
Motor neurons were analyzed 6 days after treatment with siRNAs.

### 2.3. Confocal Microscopy

Motor neurons previously seeded and differentiated onto glass coverslips were fixed with 4% paraformaldehyde for 15 min at room temperature. For C-terminal TDP-43 immunofluorescence, cells were permeabilized with a solution of 10% Triton X-100 (Serva Feinbiochemica, Heidelberg, Germany) diluted 1:5000 in PBS for 10 min, as previously described [40]. For Tuj1, a solution of 0.1% Triton X-100 in PBS was used for 10 min. After 1 h of incubation with a solution of 5% BSA (bovine serum albumin) and 0.3% Triton X-100 in PBS, the incubation with the primary antibodies (C-terminal TDP-43, 1:100, 12892-1-AP, from Proteintech, Rosemont, IL, USA or Tuj1, 1:1000, SAB4500088, from Merck Millipore, Burlington, MA, USA) was performed for 40 min at room temperature. After three washes with 0.1% saponin in PBS, cells were incubated with anti-rabbit Alexa 568-conjugated secondary antibody (1:400, A-10042, ThermoFisher Scientific, Waltham, MA, USA) or anti-rabbit Alexa 488-conjugated secondary antibody (1:400, A-21206, ThermoFisher Scientific, Waltham, MA, USA). Nuclei were stained with DAPI (4′,6-diamidino-2-phenylindole). After several washes, coverslips were mounted using Mowiol (Calbiochem-Novabiochem Corporation, La Jolla, CA, USA). For image acquisition, an LSM900 confocal scanning microscope equipped with a 63× oil-immersive objective was used (Zeiss, Oberkochen, Germany).

For the evaluation of lysosomal function, motor neurons were treated with 1 μM LysoTracker Red DND-99 (ThermoFisher Scientific, Waltham, MA, USA) for 2 h at 37 °C, then fixed, and immunolabeled as described above.

### 2.4. 2′,7′-Dichlorodihydrofluorescein Diacetate (H_2_DCFDA) Staining

H_2_DCFDA (D399, ThermoFisher Scientific, Waltham, MA, USA) was used to measure total ROS (reactive oxygen species). Cells were incubated with 10 µM H_2_DCFDA at 37 °C for 30 min. After three washes in PBS, motor neurons were lysed in 100 µL of RIPA (radioimmunoprecipitation assay) buffer (50 mM Tris-HCl, pH 8.0, with 150 mM sodium chloride, 1.0% Igepal CA-630 (NP-40) (Sigma-Aldrich, Burlington, MA, USA), 0.5% sodium deoxycholate, and 0.1% sodium dodecyl sulfate) for 5 min on ice and lysates were then centrifuged at 10,000 rpm (rotations per minutes) for 10 min at 4 °C. 90 µL of each sample were transferred to a black 96-well plate and Varioskan™ LUX Multimode Microplate Reader (ThermoFisher Scientific, Waltham, MA, USA) was used to read fluorescence at 490 nm. The remaining 10 µL of each sample were used to measure protein concentration using a BCA (bicinchoninic acid) protein assay kit (ThermoFisher Scientific, Waltham, MA, USA), allowing for normalization of fluorescence intensities based on the protein concentration of each sample.

### 2.5. Cell Viability Assay

Cell viability was measured by the sulforhodamine B (SRB) assay. Briefly, the cell medium was changed, and cells were fixed with 125 µL of 50% cold TCA (trichloroacetic acid) for 1 hour at 4 °C. After five washes with water, motor neurons were allowed to dry overnight at room temperature. 100 µL of SRB solution (0.4% SRB in 1% acetic acid) were added to each well for 30 min. After four washes with 200 µL of 1% acetic acid, SRB was solubilized by adding 800 µL of Tris 10 mM pH 10.5 per well for 10 min. Absorbance was read in a microplate reader (Victor X5, Perkin Elmer, Shelton, CT, USA) at 565 nm.

### 2.6. Western Blot

Cells were lysed in Laemmli buffer [100 mM Tris–HCl, pH 6.8, 4% (*w*/*v*) SDS, 0.2% (*w*/*v*) bromophenol blue, 20% glycerol, and 200 mM DTT (dithiothreitol)]. Proteins were blotted on PVDF (polyvinylidene) membranes (IPVH00010, Merck-Millipore, Burlington, MA, USA), which were then blocked with 5% milk in PBS and incubated with primary antibodies (mouse monoclonal anti-Hsp90, sc-13119, 1:5000, from Santa Cruz Biotechnology, Santa Cruz, CA, USA; rabbit monoclonal anti-ChAT, #27269, 1:1000, from Cell Signaling Technology, Danvers, MA, USA) overnight at 4 °C. The next day, membranes were incubated with HRP-conjugated secondary antibodies (1:5000 from Bio-Rad, Hercules, CA, USA) for 1 h at room temperature. ECL substrates Clarity or Clarity max reagents from Bio-Rad were used for the chemiluminescence reaction, and the signal was captured using ChemiDoc MP Imaging Systems (Bio-Rad, Hercules, CA, USA). Image Lab software 6.1 (Bio-Rad) was used for densitometric analysis.

### 2.7. Real-Time PCR

RNA from control and ALS iPSC-derived motor neurons was extracted using the RNeasy Plus Micro kit (74034, Qiagen, Hilden, Germany) following the manufacturer’s instructions. cDNA was synthesized from 1 μg of total RNA using SuperScript IV reverse transcriptase (18090050, Invitrogen, Waltham, MA, USA). PCR reactions were prepared with SYBR^TM^ Green PCR Master Mix (4367659, Applied Biosystem, Carlsbad, CA, USA) and run in CFX Opus 96 Thermal Cycler (Bio-Rad, Hercules, CA, USA) with the following protocol: 3 min at 94 °C; 40 cycles of 30 s at 94 °C, 30 s at 60 °C. The analysis was performed following the 2^−ΔΔCT^ method. The normalization was performed using Rplp0 (Ribosomal Protein Lateral Stalk Subunit P0).

Primer sequences are the following:

Rplp0:
forward: 5′-TCGACAATGGCAGCATCTAC-3′
reverse: 5′-ATCCGTCTCCACAGACAAGG-3′
TDP-43:
forward: 5′-GTATGATGGGCATGTTAGC-3′
wild-type reverse: 5′-CTGCACCAGAATTAGAGC-3′
mutant reverse: 5′-CTGCACCAGAATTAGAGT-3′
SOD1:
forward: 5′-GGTGGGCCAAAGGATGAAGAG-3′
reverse: 5′-CCACAAGCCAAACGACTTCC-3′
SOD2:
forward: 5′-GCTCCGGTTTTGGGGTATCTG-3′
reverse: 5′-GCGTTGATGTGAGGTTCCAG-3′
PGC1α (peroxisome proliferator-activated receptor-gamma coactivator 1-alpha):
forward: 5′-GCTGACAGATGGAGACGTGA-3′
reverse: 5′-TGCATGGTTCTGGGTACTG-3′
NRF1 (nuclear respiratory factor 1):
forward: 5′-CCGTTGCCCAAGTGAATTAT-3′
reverse: 5′-ACTGTAGCTCCCTGCTGCAT-3′
TFAM (mitochondrial transcription factor A):
forward: 5′-CCGAGGTGGTTTTCATCTGT-3′
reverse: 5′-ACGCTGGGCAATTCTTCTAA-3′
PGC1β (peroxisome proliferator-activated receptor-gamma coactivator 1-beta):
forward: 5′-TGGAAAGCCCCTGTGAGAGT-3′
reverse: 5′-TTGTATGGAGGTGTGGTGGG-3′

### 2.8. Statistical Analysis and AI Tool

The experiments were performed at least in triplicate. Data were statistically analyzed using Student’s *t*-test or one-way ANOVA followed by Dunnett’s multiple comparison tests (* = *p* < 0.05, ** = *p* < 0.01, and *** = *p* < 0.001). Graphs represent mean value ± standard error mean (SEM).

The fluorescence intensity was determined using ImageJ Software 1.54d and evaluated by quantifying the Corrected Total Cell Fluorescence (CTCF), as previously described [41]. To quantify cytoplasmic and nuclear TDP-43 fluorescence, for each cell, the nuclear region of interest (ROI) was defined using the DAPI channel and manually outlined. The cytoplasmic ROI was defined by manually outlining the entire cell based on TDP-43 signal, and subsequently subtracting the nuclear ROI to obtain a cytoplasm-specific area. The Corrected Total Cell Fluorescence (CTCF) was calculated for both nuclear and cytoplasmic compartments.

## 3. Results

### 3.1. Allele-Specific Silencing Reduces TDP-43 Mislocalization in ALS Motor Neurons

**Figure 1:**
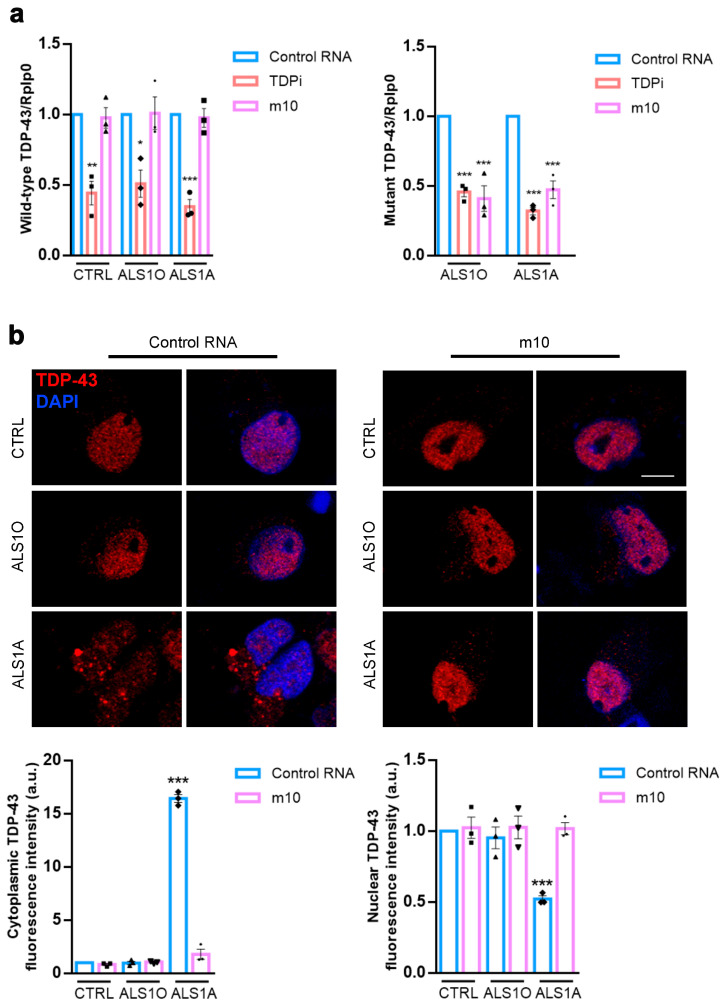
Allele-specific siRNA m10 reduces TDP-43 cytoplasmic aggregates. (**a**) Control and ALS iPSC-derived motoneurons were treated with control RNA, a siRNA targeting the 5′ region of TDP-43 mRNA (TDPi), or m10 siRNA for 6 days. The amount of wild-type and mutant TDP-43 mRNA was quantified by Real-Time PCR using Rplp0 for normalization. The graph represents the mean ± SEM of three independent experiments. Statistical analysis was performed using one-way ANOVA followed by Dunnett’s multiple comparison test, and selecting the control RNA sample as the reference sample for each group. * = *p* < 0.05; ** = *p* < 0.01; *** = *p* < 0.001. Each symbol on the bar represents an independent experiment. (**b**) Control and ALS iPSC-derived motoneurons were treated with control RNA or m10 siRNA for 6 days. Then, the cells were fixed and immunolabeled with an antibody against the C-terminal region of TDP-43, followed by an Alexa 568-conjugated secondary antibody. Nuclei were stained with DAPI. Bar = 10 µm. Nuclear and cytoplasmic fluorescence intensities were quantified as corrected total cell fluorescence using ImageJ Software 1.54d. The graph represents the mean ± SEM of three independent experiments. Statistical analysis was performed using one-way ANOVA followed by Dunnett’s multiple comparison test, comparing the same conditions (control RNA or m10) and selecting control cells as the reference sample. *** = *p* < 0.001. Each symbol on the bar represents an independent experiment.

ALS-associated mutations in TDP-43 result in the mislocalization of the protein, leading to its accumulation in the cytoplasm. Indeed, TDP-43 cytoplasmic aggregates are considered a hallmark of the disease [4]. The G376D mutation in TDP-43, which is responsible for familial ALS, also caused TDP-43 mislocalization [25,42]. We previously identified a siRNA (called m10) that specifically silences the mutated allele coding for TDP-43^G376D^, without interfering with the wild-type allele and reducing the presence of TDP-43 aggregates in transfected cells and in patient-derived fibroblasts [25]. Since the disease primarily affects motor neurons, the next step was to evaluate its efficacy in a motor neuron model derived from induced pluripotent stem cells (iPSCs). For this purpose, we selected three iPSC lines: one from a healthy individual (named CTRL), and two from a donor carrying the TDP-43 G376D mutation [38], collected at the early (ALS1O) and late stages of the disease (ALS1A). iPS cells were first nucleofected with a PiggyBac vector encoding genes involved in motor neuron differentiation. Following antibiotic selection, they were induced to mature into motor neurons. On day 12, cells were treated with either control RNA or m10, and six days later, they were processed for further analysis. First, we checked the efficacy of m10 in selectively silencing mutant TDP-43 expression in iPSC-derived motor neurons. For this purpose, we also treated cells with a siRNA targeting the 5′ region of TDP-43 (called TDPi) as a control. We performed Real-Time PCR analysis using a primer pair specific for the wild-type sequence of TDP-43 mRNA and a primer pair for the mutant sequence of TDP-43 mRNA. In both cases, we observed reduced TDP-43 mRNA levels in samples treated with TDPi, as expected, since this siRNA is able to downregulate both wild-type and mutant transcripts. Importantly, m10 downregulated only the mutant transcript of TDP-43 without affecting the wild-type mRNA (Figure 1a). This data indicates the specificity of m10 for mutant allele-specific silencing, confirming our previous results in other cellular models. Having confirmed the efficacy of the silencing protocol, we then evaluated the ability of m10 in reducing TDP-43 mislocalization in ALS motor neurons, referring to “mislocalization” as cells showing a relative loss of predominantly nuclear TDP-43 localization accompanied by increased cytoplasmic TDP-43 signal. Cells were immunolabeled with an antibody targeting the C-terminal region of TDP-43. As expected, ALS motor neurons exhibited TDP-43 mislocalization, which was absent in control cells. Notably, this phenotype was evident in ALS1A cytoplasm, unlike in ALS1O motor neurons, which are more similar to CTRL cells, indicating that predominant cytoplasmic localization of TDP-43 occurs with disease progression. Transfection with m10 effectively reduced cytoplasmic TDP-43 mislocalization in ALS1A motor neurons, rendering their phenotype comparable to that of ALS1O cells (Figure 1b).

This data indicates that m10 can counteract the primary hallmark of ALS, significantly reducing TDP-43 mislocalization and its accumulation in the cytoplasm. These changes are consistent with the rescue of cellular phenotypes observed in mutant motor neurons and indicate that m10 primarily restores normal TDP-43 localization dynamics without affecting TDP-43 protein levels.

### 3.2. Allele-Specific Silencing Ameliorates Lysosomal Function in ALS Motor Neurons

**Figure 2:**
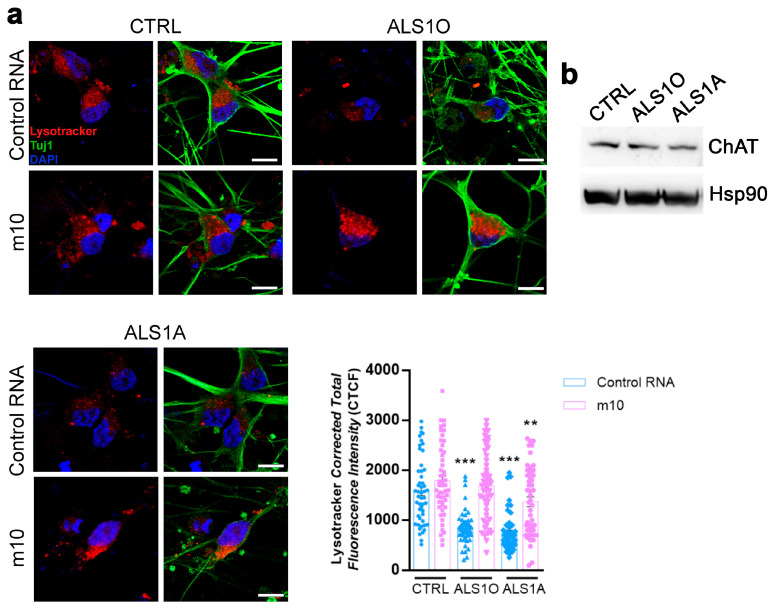
Allele-specific siRNA m10 ameliorates lysosomal functionality. (**a**) Control and ALS iPSC-derived motoneurons were treated with control RNA or m10 siRNA for 6 days. Cells were treated with 1 µM Lysotracker Red DND-99 for 2 h at 37 °C and then fixed and immunolabeled with an anti-Tuj1 antibody, followed by Alexa 488-conjugated secondary antibody. Nuclei were stained with DAPI. Bar = 10 µm. Lysotracker CTCF was calculated using ImageJ Software 1.54d. Data represent the mean ± SEM of three independent experiments. Statistical analysis was performed using one-way ANOVA followed by Dunnett’s multiple comparison test, comparing the same conditions (control RNA or m10) and selecting control cells as the reference sample. ** = *p* < 0.01, *** = *p* < 0.001. Quantification was performed on 50 cells per condition from three independent experiments. Each symbol on the bar represents one cell. (**b**) Cells were lysed and subjected to Western blot analysis using ChAT and Hsp90 antibodies.The original western blot image is shown in the Supplementary Materials Figure S1.

TDP-43^G376D^ is associated with lysosomal dysfunction. We previously demonstrated that ALS cells have more lysosomes, probably attempting to counteract their altered function. Indeed, we observed reduced lysosomal degradation activity and reduced acidification in cells transfected with a plasmid encoding TDP-43^G376D^ and in patient-derived fibroblasts carrying this mutation. Moreover, we were able to confirm lysosomal accumulation and reduced lysosomal activity in ALS iPSC-derived motor neurons [26]. Therefore, we decided to investigate whether m10 treatment could have a positive effect on lysosomal function in iPSC-derived motor neurons. To do so, after six days of silencing, we treated cells with Lysotracker Red DND-99, a fluorescent dye that labels acidic organelles. Quantifying Lysotracker fluorescence intensity, we found that both ALS1O and ALS1A cells exhibited reduced Lysotracker staining compared to control cells, while in m10-treated cells, Lysotracker fluorescence is similar to that of control cells (Figure 2a). In the context of our previous findings demonstrating lysosomal accumulation and impaired lysosomal activity in this model, these changes in LysoTracker fluorescence are consistent with alterations in lysosomal acidification and indicate that m10 treatment is able to normalize this lysosomal phenotype. Notably, iPSC-derived motor neurons express the pan-neuronal marker Tuj1 and the cholinergic motor neuron marker ChAT (Choline acetyltransferase) (Figure 2a,b). While ChAT levels were unchanged between control and ALS motor neurons, suggesting that the presence of TDP-43^G376D^ does not significantly alter motor neuron induction efficiency or differentiation purity, Tuj1 seems better organized in control motor neurons rather than in ALS motor neurons. Moreover, upon allele-specific silencing with m10, Tuj1 displays a more continuous and organized pattern along neuronal processes, suggesting a partial restoration of cytoskeletal architecture and neurite stability.

Considering that this dye accumulates based on protonation [43] and that we previously observed an accumulation of LAMP1-positive organelles with a decrease in lysosomal degradative capacity in ALS motor neurons, we can conclude that ALS motor neurons show impaired lysosomal acidification and that m10 treatment improves lysosomal functionality, indicating that allele-specific silencing has the potential to reverse this pathological phenotype as well (Figure 2).

### 3.3. Allele-Specific Silencing Decreases Oxidative Stress and Improves Cell Viability

**Figure 3:**
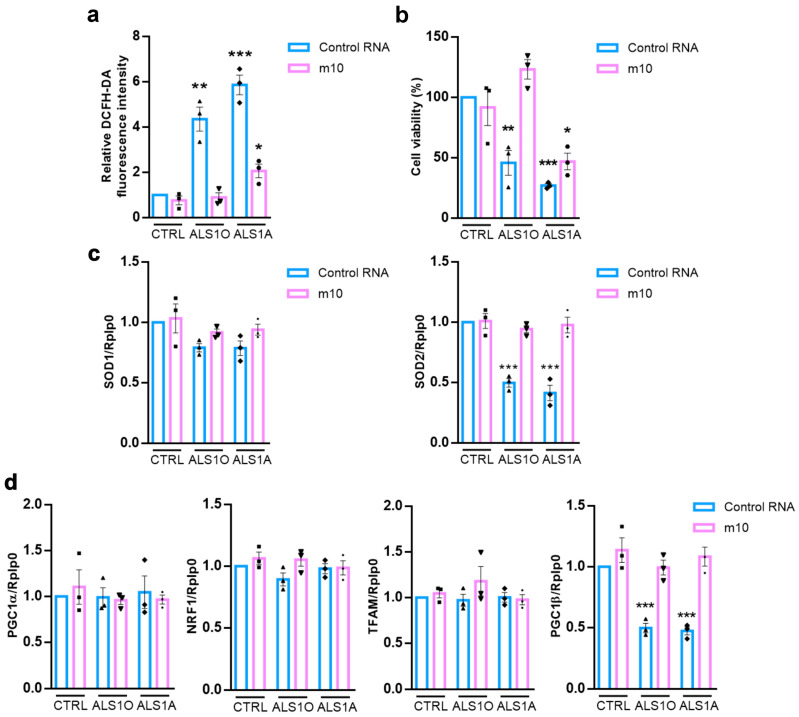
Allele-specific siRNA m10 reduces oxidative stress and increases cell viability. Control and iPSC-derived motoneurons were treated with control RNA or m10 siRNA for 6 days. Then, cells were (**a**) lysed after treatment with H_2_DCFDA, reading fluorescence intensity at 490 nm, or they were (**b**) subjected to the SRB assay, or (**c**,**d**) subjected to Real-Time PCR analysis using the indicated primers. Rplp0 was used for normalization. Data represent the mean ± SEM of three independent experiments. Statistical analysis was performed using one-way ANOVA followed by Dunnett’s multiple comparison test, comparing the same conditions (control RNA or m10) and selecting control cells as the reference sample. * = *p* < 0.05, ** = *p* < 0.01, *** = *p* < 0.001. Each symbol on the bar represents an independent experiment.

TDP-43^G376D^ is associated with mitochondrial dysfunction. Indeed, we previously observed enhanced mitochondrial localization of the mutated protein, accompanied by a decrease in the abundance of subunits from both complex I and complex II of the mitochondrial respiratory chain in transfected cells and in fibroblasts. These alterations were associated with a reduction in mitochondrial membrane potential, impaired cellular respiration, and diminished cytochrome c oxidase (COX) activity. Furthermore, ALS cells exhibited increased mitochondrial fragmentation, reduced mitochondrial biogenesis, and a decreased level of antioxidant enzymes, leading to elevated oxidative stress [27]. Therefore, we decided to evaluate oxidative stress in iPSC-derived motor neurons and the effect of m10 on it. As previously observed in transfected cells and in fibroblasts, ALS motor neurons are characterized by higher oxidative stress compared to control cells; however, when these cells were treated with m10, the production of ROS decreased (Figure 3a).

Additionally, elevated levels of ROS can impair cellular function, trigger apoptotic pathways, and drive neurodegeneration [44]. As expected, iPSC-derived ALS motor neurons exhibited reduced cell viability compared to control cells. Remarkably, treatment with m10 significantly improved their viability (Figure 3b). This effect was particularly pronounced in ALS1O motor neurons, suggesting that early-stage intervention with m10 may yield more favorable outcomes.

Having demonstrated an increased oxidative stress in ALS motor neurons, we next investigated the expression of genes involved in oxidative stress regulation: cytosolic SOD1 and mitochondrial SOD2. Under basal conditions, we observed reduced SOD2 mRNA levels in ALS cells, while SOD1 expression was unaffected (Figure 3c). Interestingly, m10 was able to restore SOD2 mRNA levels in ALS motor neurons. These data suggest that the mitochondrial antioxidant defense system is specifically impaired in ALS motor neurons and it is partially restored by mutant allele–specific silencing.

Given the observed alterations in mitochondrial antioxidant defense, we analyzed key regulators of mitochondrial biogenesis, a pathway that has been shown to be compromised in ALS fibroblasts carrying the G376D mutation in TDP-43 [27]. In basal conditions, ALS motor neurons exhibited reduced expression of PGC-1β compared to controls (Figure 3d) and m10 treatment restored its expression.

In contrast, PGC1α, NRF1, and TFAM mRNA levels were not significantly altered across conditions. These findings suggest a selective alteration of PGC-1β–dependent mitochondrial regulatory pathways in ALS motor neurons at the transcriptional level, which is ameliorated following m10 treatment.

## 4. Discussion

In this study, we demonstrated that allele-specific silencing of the mutated TDP-43^G376D^, achieved through treatment with a siRNA (designated m10), can correct several pathological phenotypes characteristic of ALS. One of these phenotypes is represented by TDP-43 mislocalization. Indeed, TDP-43 mislocalization and cytoplasmic aggregates are considered the leading pathological hallmark of the disease, with toxic gain-of-function behaviour in the cytoplasm and a loss of nuclear function [45]. In transfected cells and in ALS fibroblasts, we previously demonstrated that the G376D mutation in TDP-43 leads to the formation of cytoplasmic inclusions, and this pathological feature was reduced by the treatment with m10 [25]. Here, we show that m10 was able to specifically reduce the levels of mutant TDP-43 mRNA and treating iPSC-derived motor neurons with m10 leads to reduced cytoplasmic mislocalization of TDP-43, confirming its efficacy also in the cells primarily affected by the disease. Moreover, m10 is able to improve lysosomal function, decrease oxidative stress, and enhance cell viability in iPSC-derived motor neurons. At the transcriptional level, ALS motor neurons displayed reduced basal expression of PGC-1β and SOD2, while PGC-1α, NRF1, TFAM, and SOD1 remained unchanged. The selective reduction in PGC-1β is notable, as PGC-1β contributes to the maintenance of basal mitochondrial oxidative metabolism [46]. Notably, PGC-1β reduction is sufficient to cause mitochondrial dysfunction and oxidative stress [47]. Moreover, PGC-1α and PGC-1β have partially overlapping but non-redundant functions, and PGC-1α does not fully compensate for PGC-1β loss [48]. We previously demonstrated that PGC1α, NRF1 and TFAM were downregulated at the protein level in transfected cells and ALS fibroblasts, suggesting post-transcriptional regulatory mechanisms, consistent with the known role of TDP-43 in RNA metabolism and translational control [27]. These findings reinforce and expand upon previous data obtained in patient-derived fibroblasts, underscoring that neurons—the primary targets in ALS—may particularly benefit from this allele-specific strategy [25]. Notably, ALS1O and ALS1A cells display distinct pathological features: while ALS1A motor neurons exhibit evident TDP-43 mislocalization, this alteration is absent in ALS1O cells. This result agrees with our previous findings [25] and does not contrast with the reprogramming strategy. Indeed, reprogramming does not always fully erase somatic epigenetic features; residual epigenetic marks can persist, potentially influencing differentiation capacity and maintaining age-related or disease-related chromatin signatures [49,50]. Moreover, the patient’s systemic state (inflammation, exposure to drugs, oxidative load) at the time of biopsy can leave lasting marks (DNA damage, altered mitochondrial pool, stable chromatin changes) that re-emerge in differentiated neurons [50,51].

Lysosomal alterations, elevated oxidative stress, and reduced cell viability are shared pathological features of both ALS1O and ALS1A cells, whereas cytoplasmic TDP-43 aggregates are detected only in ALS1A cells. This observation suggests that upstream pathogenic mechanisms—such as oxidative damage, mitochondrial dysfunction, and impaired lysosomal or autophagic pathways—must exceed a critical threshold before protein aggregation becomes evident. Accordingly, early-stage cells may exhibit stress-related phenotypes in the absence of detectable insoluble aggregates [16,52]. Therefore, aggregation is often provoked by additional triggers (such as prolonged stress). The diagnosis-time cells may have early dysfunction but not yet experienced the second hit(s) required to nucleate inclusions [16,53]. Furthermore, only a small fraction of cells may form visible aggregates, and this fraction expands with time. Therefore, the difficulty in detecting aggregates could be the reason for the apparent absence of rare aggregation events in ALS1O cells.

A relevant aspect is the ability of m10 to selectively target the mutated allele, not affecting the wild-type allele. Indeed, m10 treatment leads to a clear decrease in aberrant TDP-43 mislocalization, accompanied by a more physiological distribution pattern. This indicates that m10 primarily restores normal TDP-43 localization dynamics without affecting TDP-43 protein levels. This is a crucial point, as TDP-43 levels must be within a strict range. Indeed, both an excess and a lack of the protein are detrimental to neuronal survival [54,55]. Allele-specific silencing answers to this fine-tuning need and could offer a benefit compared to the generalized downregulation of TDP-43. Importantly, total TDP-43 levels remain unchanged following m10 treatment, consistent with the known autoregulatory mechanisms controlling TDP-43 expression [37]. Thus, m10 selectively reduces the aggregation-prone mutant protein while preserving overall TDP-43 homeostasis.

Moreover, the results obtained on oxidative stress, mitochondrial biogenesis, and cell viability suggest that this type of intervention could impact not only molecular markers of the disease, such as TDP-43 aggregates, but also neuronal survival. It is worth noting that the beneficial effect of m10 is higher in ALS1O motor neurons, suggesting that early intervention could lead to a more pronounced clinical benefit. This aspect recalls the experience of other gene therapies for ALS, such as Tofersen for SOD1 mutations. Indeed, its clinical benefit is higher in patients who are treated early [56]. This leads to an important take-home message: timely genetic diagnosis and early intervention could be crucial for the success of allele-specific therapies. The discrepancy between disease onset and diagnosis represents a challenge for ALS treatment, in addition to the fact that a lot of cases show phenotypic manifestations that do not fulfill the criteria used for diagnosis, and the impossibility of finding a single cause responsible for ALS [57,58,59]. Indeed, several factors contribute to ALS onset and progression: environmental, genetic, and age-related components are responsible for the death of upper and lower motor neurons, leading to the symptoms that characterize this disorder [60]. Therefore, the transition to personalized therapy is particularly necessary for ALS, considering its heterogeneity that could explain, at least in part, the suboptimal results obtained in clinical trials [61]. Moreover, the need for an early intervention to ensure a better outcome for patients makes it essential to identify biomarkers that offer the possibility to not only accelerate ALS diagnosis but also to help in prognostic evaluation. Promising results have been achieved with a combination of neurofilament L (NfL) and miRNA-181 [62].

Currently, three compounds are used for ALS treatment: Riluzole, Edavarone, and Sodium phenylbutyrate/Taurursodiol [28,30,63]. The administration of these drugs slows disease progression, increasing survival by a few months [64,65]. Moreover, as it is estimated that the number of ALS patients worldwide will be about 380,000 by 2040, new treatment options are necessary [66]. The mechanism of action of these medications is based on targeting oxidative stress and excitotoxicity, but other pathways are involved in ALS development, such as lysosomal dysfunction [67]. Therefore, targeting other pathways could lead to greater benefits for patients. With the data presented in this paper, we demonstrated that m10 could offer this possibility, as it not only reduces oxidative stress but also increases lysosomal functionality, thereby reducing the presence of TDP-43 cytoplasmic aggregates and increasing cell viability.

Despite the promising results, this study has some limitations. The data were obtained from an in vitro model, and it will be necessary to validate the efficacy of the siRNA in living organisms. Moreover, the primary challenge remains in vivo delivery, as achieving safe, stable, and efficient delivery to motor neurons is a complex task. Several platforms, including viral vectors (AAV) and lipid nanoparticles, are currently under investigation for clinical applications and could be adapted to our siRNA [68]. It will also be essential to assess the duration of the therapeutic effect and the potential need for repeated administrations. Additionally, further studies will be focused on the evaluation of the effectiveness of m10 in reversing other cellular alterations linked to the TDP-43^G376D^ mutation, such as mitochondrial dysfunctions.

## 5. Conclusions

Our findings support the notion that allele-specific silencing through siRNA represents an innovative and promising approach for ALS caused by the TDP-43^G376D^ mutation. siRNA treatment can revert some ALS-associated pathological phenotypes, such as TDP-43 mislocalization, lysosomal dysfunctions, and oxidative stress in iPSC-derived motor neurons, confirming the data previously obtained in other cellular models. An early treatment seems fundamental to achieve better outcomes for patients, given the results obtained in ALS1O motor neurons compared with ALS1A. This also highlights the need for biomarkers for early diagnosis. If confirmed by further preclinical and clinical studies, siRNA treatment could pave the way for a new class of personalized therapies for ALS, based on the selective correction of disease-causing genetic mutations.

## Data Availability

The original contributions presented in the study are included in the results. Further inquiries can be directed to the corresponding author.

## Supplementary Materials

The following supporting information can be downloaded at: https://www.mdpi.com/article/10.3390/biom16030393/s1, Figure S1: The original western blot image of Figure 2b.

## Abbreviations

The following abbreviations are used in this manuscript:

ALS	amyotrophic lateral sclerosis
BCA	bicinchoninic acid
BSA	bovine serum albumin
C9orf72	chromosome 9 open reading frame 72
ChAT	Choline acetyltransferase
COX	cytochrome c oxidase
CTF	C-terminal fragment
DAPI	4′,6-diamidino-2-phenylindole
FUS	fused in sarcoma
H2DCFDA	2′,7′-Dichlorodihydrofluorescein diacetate
iPSC	induced pluripotent stem cell
LAMP1	lysosomal associated membrane protein 1
NRF1	nuclear respiratory factor 1
PBS	phosphate-buffered saline
PGC1α	peroxisome proliferator-activated receptor-gamma coactivator 1-alpha
PGC1β	peroxisome proliferator-activated receptor-gamma coactivator 1-beta
RIPA	radioimmunoprecipitation assay
ROS	reactive oxygen species
Rplp0	Ribosomal Protein Lateral Stalk Subunit P0
rpm	rotations per minutes
siRNA	small interfering RNA
SOD1	superoxide dismutase 1
SRB	sulforhodamine B
TARDBP	TAR DNA-binding protein
TCA	trichloroacetic acid
TFAM	mitochondrial transcription factor A
