# Supplementary figures and images for: Selective Silencing of TDP-43 P. G376D Mutation Reverses Key Amyotrophic Lateral Sclerosis-Related Cellular Deficits
Source: Biomolecules. 2026 Mar 5;16(3):393. doi: 10.3390/biom16030393 (PMC13024069; doi:10.3390/biom16030393)

**Figure S1: The original western blot image of Figure 2b**

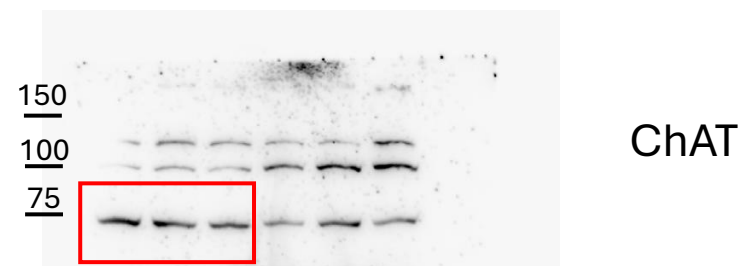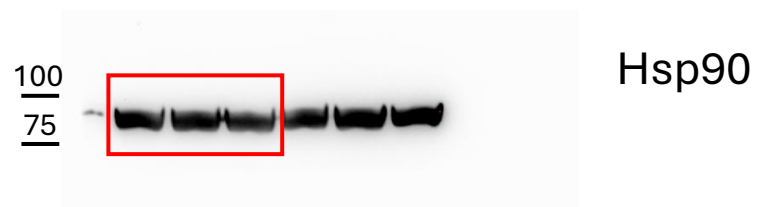

Supplement: Supplementary file 1 [file biomolecules-16-00393-s001.zip › biomolecules-4050949-supplementary.pdf]
